# Advanced thymic carcinoma with suspected cardiac metastasis: multimodal diagnostic precision using CT, PET-CT, and immunohistochemistry

**DOI:** 10.11604/pamj.2025.52.158.49755

**Published:** 2025-12-15

**Authors:** Juan Carlos Perdomo Puentes, Bianca Cristea

**Affiliations:** 1 Francisco Gentil, Portuguese Institute of Oncology of Lisbon (IPO), Lisbon, Portugal

**Keywords:** Thymic carcinoma, anterior mediastinal mass, cardiac metastasis, imaging diagnosis, palliative care

## Image in medicine

A 59-year-old woman with no prior history of malignancy presented with progressive chest pain, anorexia, fatigue, and unintentional weight loss. Chest radiography revealed a large anterior mediastinal opacity. Contrast-enhanced computed tomography (CT) demonstrated a heterogeneous anterior mediastinal mass measuring 73 × 67 × 62 mm, with deep calcifications and direct invasion of the pericardium and main pulmonary artery, associated with a 13 mm pericardial effusion. Enlarged mediastinal lymph nodes were observed in the prevascular, paratracheal, and subcarinal regions. Positron emission tomography (PET) showed intense hypermetabolic activity in the mediastinal mass and lymph nodes, as well as focal uptake in the cardiac region, increasing the suspicion of myocardial involvement. Pulmonary micronodules and adrenal lesions showed no significant metabolic activity. A percutaneous biopsy guided by computed tomography was performed, and histopathological examination revealed atypical polygonal epithelial cells and frequent mitoses. Immunohistochemical analysis was positive for CD5, CD117, p63, cytokeratin AE1/AE3, and CAM5.2, and negative for PAX8 and CK7, excluding pulmonary and thyroid origins. These findings established the diagnosis of advanced thymic epithelial carcinoma of the anterior mediastinum, with pericardial invasion and suspected cardiac involvement. Due to the advanced stage of the disease and poor prognosis, curative oncological treatment was not performed. The approach focused on palliative care, with pain control, treatment of dyspnea, and support for fatigue and functional decline. This case highlights the importance of multimodal imaging and immunohistochemistry in the diagnosis and staging of thymic carcinoma, and underscores the essential role of early palliative care in advanced mediastinal neoplasms.

**Figure 1:**
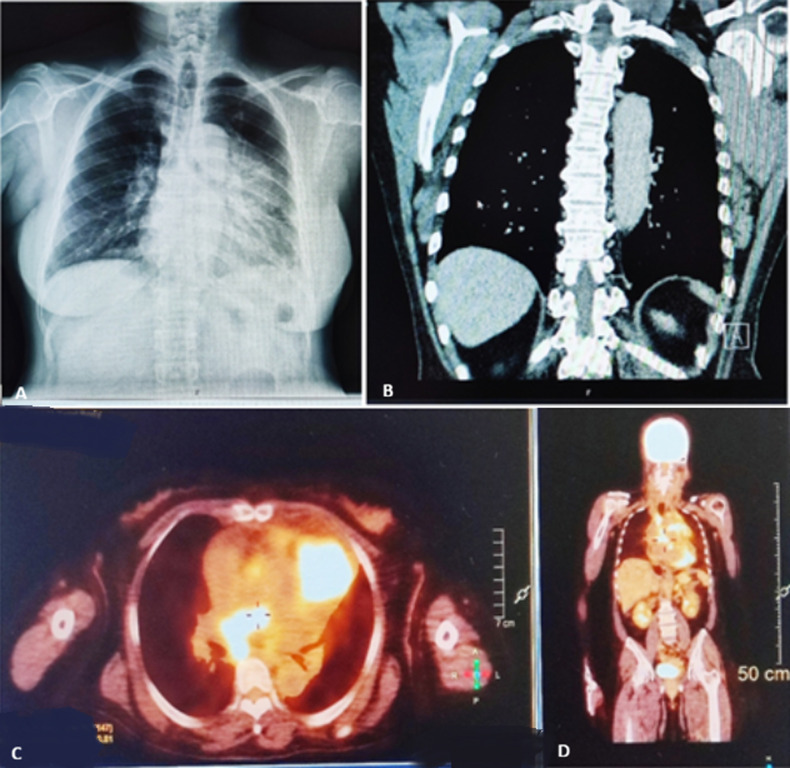
(A,B,C,D) multimodal imaging of advanced thymic carcinoma

